# The Effect of Tai Chi Training on Cardiorespiratory Fitness in Healthy Adults: A Systematic Review and Meta-Analysis

**DOI:** 10.1371/journal.pone.0117360

**Published:** 2015-02-13

**Authors:** Guohua Zheng, Shuzhen Li, Maomao Huang, Feiwen Liu, Jing Tao, Lidian Chen

**Affiliations:** 1 College of Rehabilitation Medicine, Fujian University of Traditional Chinese Medicine, Fuzhou, China; 2 Fujian University of Traditional Chinese Medicine, Fuzhou, China; University Of São Paulo, BRAZIL

## Abstract

**Background:**

Tai Chi may be efficient for healthy adults to improve the cardiorespiratory fitness, but there is no systematic evaluation for its effectiveness.

**Objective:**

To systematically assess the effectiveness of Tai Chi on cardiorespiratory fitness in healthy adults.

**Methods:**

Seven electronic databases were searched from their inception to October 2013. The controlled trails including randomized controlled trial (RCT), non-randomized controlled trial (NRCT), self-controlled trial (SCT), and cohort study (CS) testing Tai Chi exercise against non-intervention control conditions in healthy adults that assessed any type cardiorespiratory fitness outcome measures were considered. Two reviewers independently performed the selection of the studies according to predefined criteria. The risk of bias was assessed using Cochrane criteria. RevMan 5.2 software was applied for data analysis.

**Results:**

Twenty studies (2 RCTs, 8 NRCTs, 3 SCTs, and 7 CSs) with 1868 participants were included, but most of them belonged to low methodological quality. The results of systematic review showed that Tai Chi exercise had positive effect on majority outcomes of cardio function (Blood pressure: n = 536, SPB SMD = -0.93, 95% CI -1.30 to -0.56, P < 0.00001; DBP SMD = -0.54, 95% CI -0.90 to -0.18, P < 0.00001; heart rate at quiet condition: n = 986, SMD = -0.72, 95% CI -1.27 to -0.18, P = 0.010; stroke volume: n = 583, SMD = 0.44, 95% CI 0.28 to 0.61, P < 0.00001; cardio output: n = 583, MD = 0.32 L/min, 95% CI 0.08 to 0.56, P = 0.009), lung capacity (FVC at quiet condition: n = 1272, MD = 359.16 mL, 95% CI 19.57 to 698.75, P = 0.04 for less than one year intervention, and MD = 442.46 mL, 95% CI 271.24 to 613.68, P<0.0001 for more than one year intervention; V·O_2_peak: n = 246, SMD = 1.33, 95% CI 0.97 to 1.70, P < 0.00001), and cardiorespiratory endurance (O_2_ pulse at quiet condition: n = 146, SMD = 1.04; 95% CI 0.69 to 1.39; P < 0.00001; stair test index at quiet condition: n = 679, SMD = 1.34, 95% CI 0.27 to 2.40, p = 0.01). No adverse events were reported.

**Conclusions:**

The results are encouraging and suggest that Tai Chi may be effective in improving cardiorespiratory fitness in healthy adults. However, concerning the low methodological quality in the included studies, more larger-scale well-designed trails are needed till the specific and accurate conclusions can be perorated.

## Introduction

Cardiorespiratory fitness (CRF) is a measure of the capacity of the cardiovascular system to transport oxygen and the ability of the muscles to use it. Furthermore CRF indicates the ability of the circulatory, respiratory, and muscular systems to supply oxygen to skeletal muscles during sustained physical activity [1]. It therefore is strongly associated with various health outcomes. Substantial data have demonstrated that CRF is associated with morbidity and mortality in general population independently of other risk factors [2–4]. Compared with the individuals with the low level CRF, those with high level CRF had 53% lower risk for all-cause mortality for women and 43% for men [5]. Moreover, the efficient CRF has benefit to reduce the risk of heart disease, lung cancer, type 2 diabetes, stroke, and other chronic diseases [6–7]. However, with increasing of age in the general population, cardiorespiratory system generates certain changes in shape and functionality, which shows the angiosclerosis of arterial vascular walls, the decline of blood vessel compliance, enlargement of peripheral resistance, increase of blood pressure, decline of respiratory muscle function and low-efficiency work of breathing [8]. Thus low CRF is an important risk factor for the development of cardiovascular disease. CRF also is an independent predictor of mortality, and provides significantly more accurate prognostic information [9].

Although CRF has a genetic contribution, physical activity habits are its primary determinant in adults, and changes in physical activity levels result in changes in CRF [10–12]. Furthermore intensity and duration of physical activity may emerge as the best independent predictor of CRF [13–14]. A number of published studies have demonstrated that regular physical activity can significantly increase aerobic capacity as well as lung capacity and decreased heart rate [15–17]. Previous studies also testified that physical activity and exercise training resulted in significant cardiorespiratory fitness improvements not only in healthy populations [18] but also in patients of traumatic brain injury [19].

Tai Chi (TC), also known as Tai Chi Chuan, developed originally as a martial art from the 17th century, and has been practiced for centuries in China as a no-contact, low-impact, soft body and mindfulness exercise for health promotion in general population [20–21]. The basic action of TC is “a series of individual movements linked together a continuous manner that flow smoothly from one movement to another” [22]. The practitioners were required to keep deep diaphragmatic and rhythmic breathing, and integrate into body motions to harmonize the body balance and mental concentration when TC is practiced [23]. It’s also principled upon breathing and neuromuscular active relaxation with slow and gentle movement meanwhile maintaining stable postures [24]. Studies have been performed to examine the effect of TC exercise on the physical condition of a wide age range [25–26] particularly in the elder people [27–28]. Beneficial improvement has been reported on blood pressure, immune capacity, mental control, flexibility, and balance control [29–30]. Currently, the increasing data from clinical trials and exercise intervention studies found that TC exercise associated with the improvement of CRF in both healthy people and patients with chronic diseases. For example, some studies indicated that TC training with low to moderate intensity is of great positive importance on outcomes of CRF including the oxygen uptake (Vo_2_), O_2_ pulse [31], ventilatory efficiency [32], lung function [33–35], blood pressure [36], aerobic endurance [37] and exercise capacity [38]. The results from a meta-analysis and its update suggested that Tai Chi might be effective in improving the aerobic capacity of CRF outcomes among sedentary adults with over 55 years old [39–40], whereas another previous systematic review found no significant differences [41]. Furthermore those previous review or meta-analysis did not involve in other outcomes of CRF. Therefore the convincing evidence of Tai Chi on improving CRF in the general healthy population or patients with chronic diseases was yet unclear. To our knowledge, no systematic reviews have evaluated the effect of Tai Chi exercise on the outcomes of CRF in healthy adults. We therefore designed this systematic review to investigate the effectiveness and safety of Tai Chi exercise on the outcomes of CRF in healthy adults.

## Methods

### Eligibility Criteria

Available human clinical or community studies with a randomized controlled, non-randomized controlled, self-control trials and cohort design published in English or Chinese were included in this review. The participants were defined to healthy adults. The included studies should focus on the effect of TC exercise for CRF comparing with non-intervention, which those comparing TC with other exercise intervention were excluded. Outcomes measures for CRF assessment should cover at least one of essential outcomes such as heart rate, stroke volume, cardiac output, myocardial oxygen consumption, left heart energy effective utilization, left ventricular effective pump power, gas exchange rate, forced vital capacity, forced vital capacity in the first second, maximal minute ventilation, peak oxygen uptake, myocardial oxygen consumption, oxygen pulse, expansion coefficient of elasticity of blood vessels.

### Data Sources and Searches

The original research articles were searched from 7 electronic English and Chinese databases which covered PubMed, Science Citation Index (SCI), EMBASE, Cochrane library, Chinese Scientific Journal Database (VIP), China National Knowledge Information database (CNKI) and Wan Fang from their inception to October 2013. We used the following search strategy ((*Taiji* OR *Tai Chi* OR *Chi*, *Tai* OR *Tai Ji Quan* OR Ji Quan, Tai OR *Quan*, *Tai Ji* OR *Tai-ji* OR *Taijiquan* OR *T’ai Chi* OR *Tai Chi Chuan)* AND (*cardiorespiratory function* OR *maximal oxygen* OR *FVC* OR *Forced Vital Capacity* OR *gas exchange rate* OR *stroke volume* OR *VE* OR *minute ventilation* OR *minute respiratory volume* OR *EWK* OR *myocardial oxygen consumption OR HOV* OR *MOCI* OR *HOI* OR *maximal oxygen consumption* OR *FEK* OR *expansion coefficient of elasticity of blood vessels* OR *heart rate* OR *blood pressure* OR *oxygen pulse)* AND (*control* OR *comparison* OR *controlled trial*)) in the English databases and (*Taiji* AND *cardiorespiratory function* AND *comparison*) in the Chinese databases. The detail of search strategies can be found in S1 File. The reference lists of identified articles were also searched.

### Study Identification and Quality Assessment

Two reviewers (LSZ and HMM) independently screened the title and abstract of the searched studies. Full text of the studies that potentially met the eligibility criteria were obtained, and the potentially relevant references were retrieved according to predefined eligibility criteria. Data was extracted by one reviewer (LSZ) using the prepared forms and checked for accuracy by the second (HMM). The extracted details from eligible literatures included the language of publication, age of participants, methodological information, demographics of participants, sample size, experimental and control interventions, duration, frequency and style of TC exercise, outcomes, and adverse effects. Disagreements were resolved through discussion, and the original author was contacted with if the results could not come to an agreement.

Quality of studies was assessed independently by two reviewers (LSZ and LFW) using the Cochrane Collaboration’s tool for assessing the risk of bias [42]. The following recommended domains were considered: selection bias (random sequence generation and allocation concealment), performance bias (blinding of participants and personnel), detection bias (blinding of outcome assessment), attrition bias (incomplete outcome data), reporting bias (selective reporting) and other bias, each of which was rated according to the level of bias and categorized into either low, high, or unclear.

### Data Analysis

Statistical heterogeneity among studies was assessed using a Chi-square test, or by calculating Higgins *I* ^*2*^ values [43]. The results were pooled using a fixed effect model when the *I* ^*2*^ value was less than 40%. Otherwise, a random effect model was applied. However if the *I* ^*2*^ value among studies was more than 75%, the heterogeneity was considered substantive and the overall meta-analysis was not appropriate to conducted but subgroup analysis was considered to measure the pooled effect. Sensitivity analysis was used to explore the source of heterogeneity. The mean difference (MD) or standard mean difference (SMD) with corresponding 95% confidence interval (CI) was calculated for the continuous outcomes. Review Manager 5.2 software of The Cochrane Collaboration was applied for data analysis and all *P* values were two sided [44].

## Results

### Description of Studies

**Fig 1:**
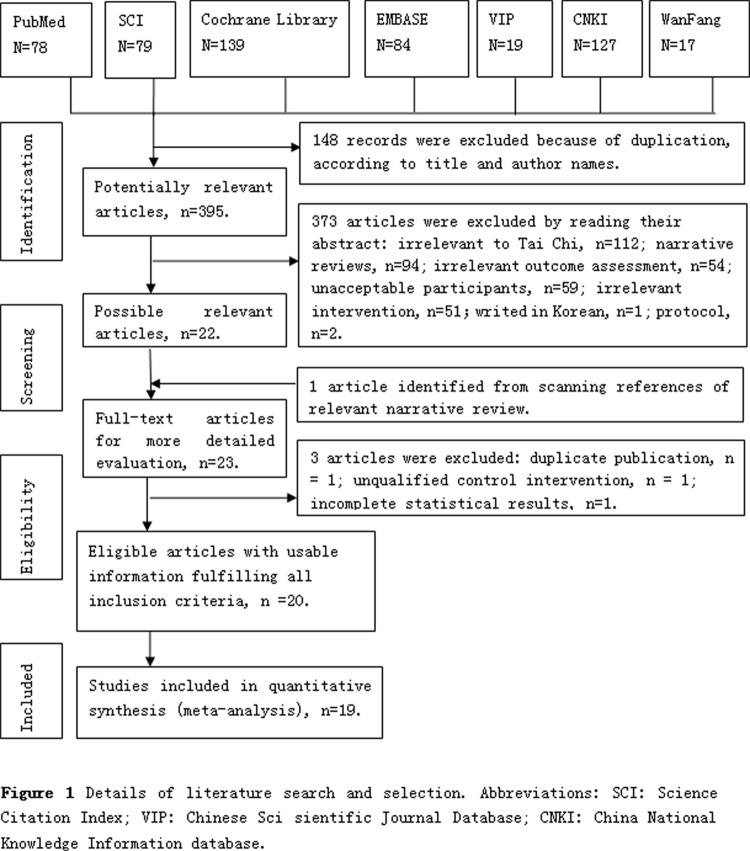
Details of literature search and selection. Abbreviations: SCI: Science Citation Index; VIP: Chinese Sci sientific Journal Database; CNKI: China National Knowledge Information database.

The detail screening flow about the generating of eligible articles was presented in the Fig. 1. A total of 532 records were identified through database searches. After removing duplicates, 395 remained to be screened for eligibility, and 23 were selected for a full-text evaluation. Of these, 3 studies were excluded, and reasons included: duplicate publication (n = 1) [45]; unqualified control intervention (n = 1) [46], and incomplete statistical results (n = 1) [47] (S2 File). Consequently, 20 literatures met with the inclusion criteria and 19 studies were included into meta-analysis.

Table 1 illustrated the characteristics of the sample, intervention and outcomes. This review involved a total of 1783 healthy community-dwellers (age from 45 to 75 years old). And the design type of those included studies covered 2 randomized controlled trials (RCTs) [48–49], 8 non- randomized controlled trials (NRCTs) [50–57], 7 Cohort studies (CSs) [58–64] and 3 Self-controlled trials (SCTs) [65–67]. Few studies [49, 54, 56, 57, 63, 64] definitely described the type of Tai Chi used in their studies. Apart from the 3 Self-controlled trials, the remaining 17 trials compared TC with non-intervention. Moreover, all the measurement of outcomes utilized acknowledged apparatus and method. Additionally, TC has not been reported to be associated with adverse events during the intervention.

**Table 1:** Characteristics of the included studies.

Author, year	Study design	Place of study	No.of participants (T/C)	Age (year)		Intervention		Frequency and Duration of Tai Chi exercise	Outcomes
				Tai Chi	Control	Tai Chi	Control		
**Nguyen et al. 2012 (48)**	RCT	Vinh, Vietnam	96(48/48)	69.23±5.3	68.73 ±4.95	Tai Chi	Non-intervention	60 minutes per time and twice per week for 6 months	HR,SBP,DBP
**Lu et al.2012(49)**	RCT	Taiwan, China	50(25/25)	50–67	47–59	Yang’s Tai Chi Chung	Non-intervention	40 minutes per time and 7 times per week for 3 months	FVC,FEV_1_,SBP,DBP
**Tu HL 2005 (50)**	NRCT	Hubei, China	32(16/16)	61.64±4.21	63.25±6.05	Taijiquan	Non-intervention	12 minutes per time and 3 times per week for 10 weeks	SV,CO,VPE,EWK,HOV,SBP,DBP
**Xu and Wen 1997 (51)**	NRCT	Sichuan, China	34(18/16)	66.7±7.4	64.6±3.9	Taijiquan	Non-intervention	for 10.5±8.7 years.	HR.SBP,DBP,MMV,FVC
**Liu and Jin 2010(52)**	NRCT	Jiangsu, China	20(10/10)	61.2±3.0	62.5±2.4	Taijiquan	Non-intervention	60 minutes per time and 4 times per week for 8 weeks.	HR.SBP,DBP,MMV,FVC,FEV1
**Yang and Fu 2010 (53)**	NRCT	Gansu, China	60(30/30)	50–70	50–70	Taijiquan	Non-intervention	35 minutes per time and more than 4 times per week for 1 year.	SBP,DBP,HR,FVC, Step test index
**Lan et al. 1998 (54)**	NRCT	Taiwan, China	39(21/18)	60.05±4.48	66±3.85	Yang’s Tai Chi Chung	Non-intervention	1 hour and 4.6 ± 1.3 times per week for 11.2 ± 1.4 months.	HR,VO_2peak_;O_2_ pulse, MMV
**Chang et al.2013 (55)**	NRCT	Taiwan, China	133(64/69)	56.45±8.51	62.26±12.91	Tai Chi Chung	Non-intervention	60 minutes per time and three times per week at a park for 12 weeks.	SBP,DBP
**Thornton et al. 2004(56)**	NRCT	UK	40(20/20)	47.2±4.07	48.4±4.33	Yang’s Tai Chi	Non-intervention	three times per week for 12 weeks.	SBP,DBP
**Lu et al.2003 (57)**	NRCT	Taiwan, China	40(20/20)	52.8±7.5	56.3±8.5	Yang’s Tai Chi Chuan	Non-intervention	40 minutes per time and 3 times per week for an average of 1.9±1.0 years。	SBP,DBP
**Rong et al. 2009 (58)**	CS	Beijing, China	421(212/209)	Over 45	Over 45	Taijiquan	Non-intervention	More than 30 minutes per time and more than 4 times per week for more than 5 years.	FVC, Step test index
**Yan Y 2013 (59)**	CS	Liaoning, China	98(48/50)	over 45	over 45	Taijiquan	Non-intervention	More than 30 minutes per time and more than 3 times per week for 1year.	FVC, Step test index
**Li XH 2008(60)**	CS	Liaoning, China	60(30/30)	65.3±4.8	66.1±4.6	Taijiquan	Non-intervention	From 40 to 60 minutes per time and more than 4 times per week for 1 years.	FVC,HR,SV,CO
**Peng CZ 2006 (61)**	CS	Zhejiang, China	380(180/200)	61.94±3.64	62.07±3.16	Taijiquan	Non-intervention	More than 30 minutes per time and 3 times per week for 5 years	FVC,HR,SV,CO,MMV
**Liang YW 2001(62)**	CS	Guangdong, China	33(15/18)	66.7±7.4	64.6 ± 3.9	Taijiquan	Non-intervention	No description	FVC,HR,SV,CO,MMV,SBP,DBP
**Lai et al.1995 (63)**	CS	Taiwan, China	84(45/39)	62.6±6.1	63.1±4.75	Yang’s Tai Chi Chuan	Non-intervention	54 minutes per time and 5 times per week for 2 years.	HR,VO_2peak_ O_2_ pulse, MMV
**Lan et al. 2004 (64)**	CS	Taiwan, China	24(12/12)	58.8±7.9	59.9±5.2	Yang’s Tai Chi Chuan	Non-intervention	54 minutes per time and 3 times per week for 4.7±3.3 years.	HR,VO_2peak_ O_2_ pulse, MMV
**Lei et al.2001 (65)**	SCT	Fujian, China	39	6I.87±4.4		Taijiquan.		30 minutes per time and 7 to 14 times per week for 1 year.	SV,CO,VPE,EWK,HOV,SBP,DBP
**Han YZ 2010 (66)**	SCT	Jilin, China	50	61.1±6.14		Taijiquan		No less than 1 hour per time and mean 4 times per week for 6 months	HR,SBP,DBP,FVC
**Wang GJ 2010 (67)**	SCT	Jilin, China	50	63.5±2.8		Taijiquan		No description	SBP,DBP,HR,FVC,VO_2peak_,Step test index

Abbreviations: RCT, randomized controlled trial; NRCT, non-randomized controlled trial; CS, Cohort study; SCT, Self-control trail; SBP, systolic blood pressure; DBP, diastolic blood pressure; HR, heart rate; SV, stroke volume; CO, cardiac output; HOV, myocardial oxygen consumption; EWK, left heart energy effective utilization; VPE, left ventricular effective pump power; FVC, forced vital capacity; FEV_1_, forced vital capacity in the first second; MMV maximal minute ventilation; VO_2peak_, peak oxygen uptake; O_2_ pulse, oxygen pulse.

### Methodological Quality

Of 2 included RCT, one trial described the random sequence generation by using the coin, and another utilized the blinding of outcome assessment. The remaining 18 trials were designed as non- randomized control, Self-control trial and cohort study. 3 studies indicated no dropouts. 4 studies reported dropouts as well as withdraw, and described their numbers and reason in detail. In all, all included studies were considered high risk of bias. The detail of risk of bias was illustrated in Table 2.

**Table 2:** The risk of bias about included studies.

Author, year	Random sequence generation (selection bias)	Allocation concealment (selection bias)	Blinding of participants and personnel (performance bias)	Blinding of outcome assessment (detection bias)	Incomplete outcome data (attrition bias)	Selective reporting (reporting bias)	Other bias	Total bias
**Nguyen et al. 2012 (48)**	Unclear	Unclear	High	High	Unclear	High	Unclear	High
**Lu et al.2012 (49)**	Low	Low	High	High	Unclear	High	Unclear	High
**Tu HL 2005 (50)**	High	High	High	High	Unclear	High	Unclear	High
**Xu and Wen 1997 (51)**	High	High	High	High	Unclear	High	Unclear	High
**Liu and Jin 2010 (52)**	High	Unclear	High	Unclear	Unclear	High	Unclear	High
**Yang and Fu 2010 (53)**	High	High	High	High	Unclear	High	Unclear	High
**Lan et al. 1998 (54)**	High	High	High	Unclear	Unclear	High	High	High
**Chang et al.2013 (55)**	High	High	High	Low	High	Unclear	Unclear	High
**Thornton et al. 2004 (56)**	High	High	High	Low	Unclear	Low	Unclear	High
**Lu et al.2003 (57)**	High	High	High	High	Unclear	High	Unclear	High
**Rong et al. 2009 (58)**	High	High	High	High	Unclear	Low	Unclear	High
**Yan Y 2013 (59)**	High	High	High	High	Unclear	High	Unclear	High
**Li XH 2008 (60)**	High	High	High	High	High	Unclear	Unclear	High
**Peng CZ 2006 (61)**	High	High	High	High	Unclear	High	Unclear	High
**Liang YW 2001 (62)**	High	High	High	High	Unclear	High	Unclear	High
**Lai et al.1995 (63)**	High	High	High	Unclear	Unclear	High	High	High
**Lan et al. 2004 (64)**	High	High	High	Unclear	Unclear	High	Unclear	High
**Lei et al.2001 (65)**	High	High	High	High	High	High	Unclear	High
**Han YZ 2010 (66)**	High	High	High	High	Unclear	Unclear	Unclear	High
**Wang GJ 2010 (67)**	High	High	High	High	Unclear	High	Unclear	High

### Measures of Effect

**Fig 2:**
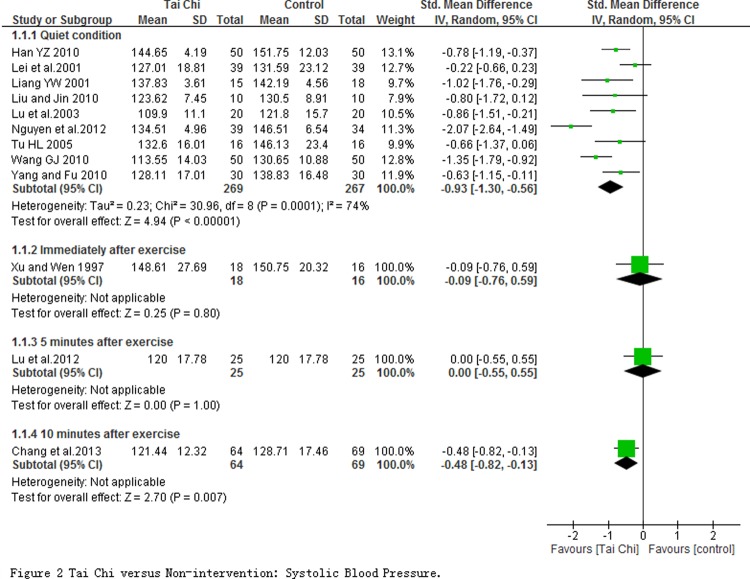
Tai Chi versus Non-intervention: Systolic Blood Pressure.

**Fig 3:**
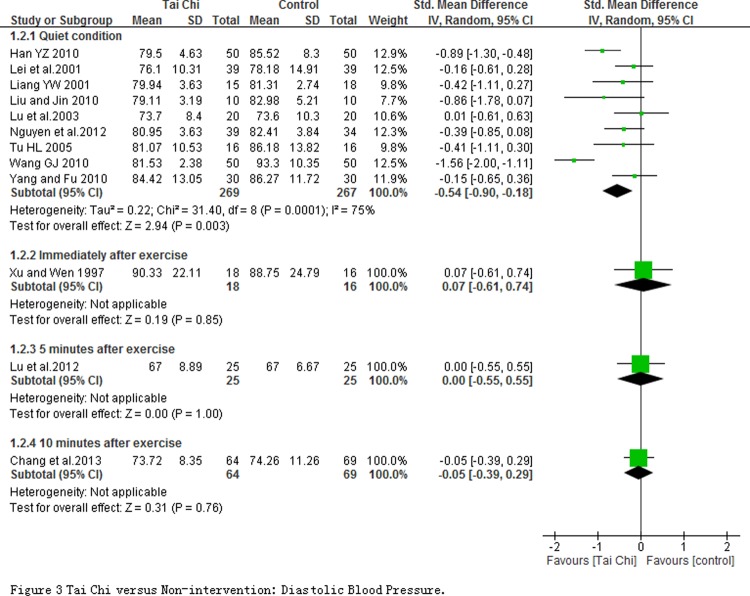
Tai Chi versus Non-intervention: Diastolic Blood Pressure.

**The effect of cardiovascular efficiency.** 12 studies involving in 753 participants reported the systolic and diastolic blood pressures which were measured at quiet condition and at immediately as well as 5 and 10 minute after exercise. Meta-analyses including 9 studies with 536 subjects revealed that SPB (SMD = -0.93; 95% CI-1.30 to-0.56; *P* < 0.00001; Fig. 2) and DBP (SMD = -0.54, 95% CI-0.90 to-0.18, *P* < 0.00001; Fig. 3) in TC exercise group significantly decreased at quiet condition comparing with non-intervention group. But significant changes on SBP or DBP between groups were not observed at immediately, 5 and 10 minutes after exercise except for the SBP at 10 minute. Moreover, one non-randomized study reported that the blood pressure had a significant decrease regardless of systolic or diastolic blood pressure after 12 weeks TC training, whereas no obvious change was found in the control group [68].

**Fig 4:**
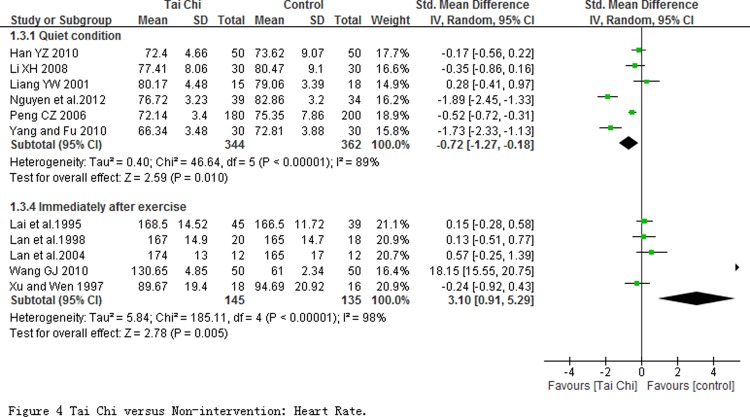
Tai Chi versus Non-intervention: Heart Rate.

11 studies with 986 participants reported heart rate outcome which was measured at quiet condition in 6 studies and immediately after exercise in other 5 studies. Of 6 studies with HR measured at quiet condition, the pooled result of meta-analysis showed a significant decline (SMD = -0.72; 95% CI-1.27, -0.18; P = 0.010; Fig. 4). But heterogeneity among studies was substantive with *I* ^2^ = 89%, and not obviously changed after sensitivity analysis by removing any one of those studies. No study but one (Wang 2010) was found a significant effect on heart rate at immediately measured after exercise. But pooled analysis showed a significant increase (SMD = 3.10; 95% CI 0.91 to 5.29) with a substantive heterogeneity (*I* ^*2*^ = 98%, *P* = 0.005). The pooled result was changed as SMD being 0.13 (95% CI 0.17 to 0.42) with low heterogeneity (*I* ^*2*^ = 0%, *P* = 0.4) after sensitivity analysis was conducted by removing Wang 2010.

**Fig 5:**
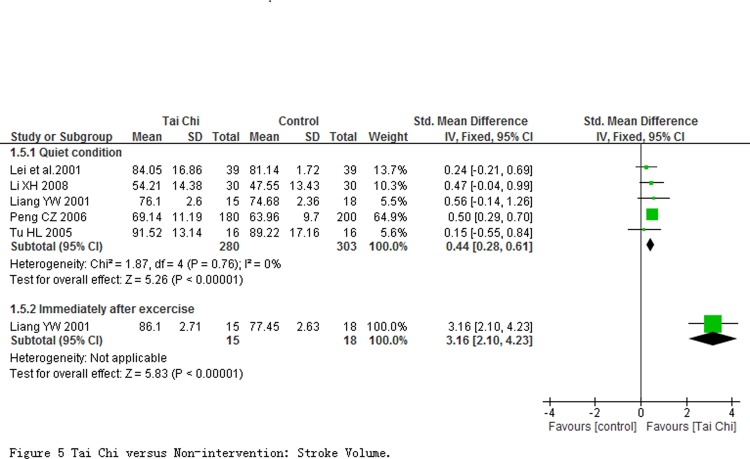
Tai Chi versus Non-intervention: Stroke Volume.

**Fig 6:**
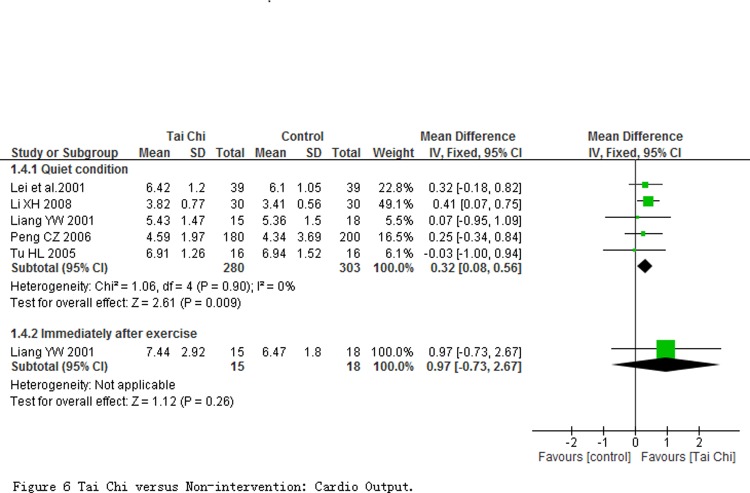
Tai Chi versus Non-intervention: Cardio Output.

Stroke volume (SV) and cardio output (CO) were evaluated at quiet condition in 5 studies involving 583 participants, and were immediately evaluated after exercise in one study with 33 participants. Comparing to non-intervention, results of meta-analyses revealed that SV and CO in participants with TC exercise at quiet condition had a significant improvement with the pooled SMD of SV being 0.44 (95% CI 0.28 to 0.61; *P* < 0.00001; Fig. 5) and pooled MD of CO being 0.32 L/min (95% CI 0.08 to 0.56; *P* = 0.009; Fig. 6), respectively. One study reported a significant improvement in SV other than CO at immediately evaluated after exercise.

**Fig 7:**
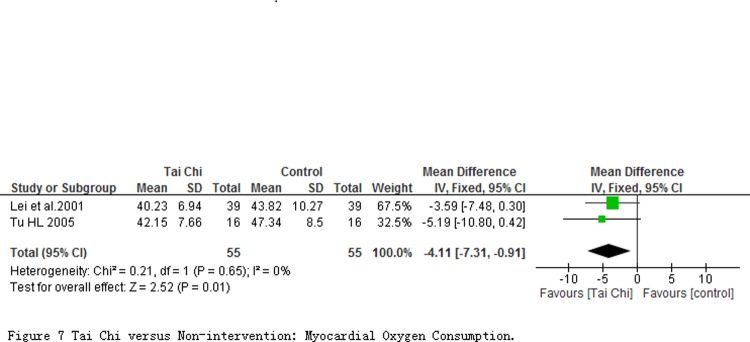
Tai Chi versus Non-intervention: Myocardial Oxygen Consumption.

**Fig 8:**
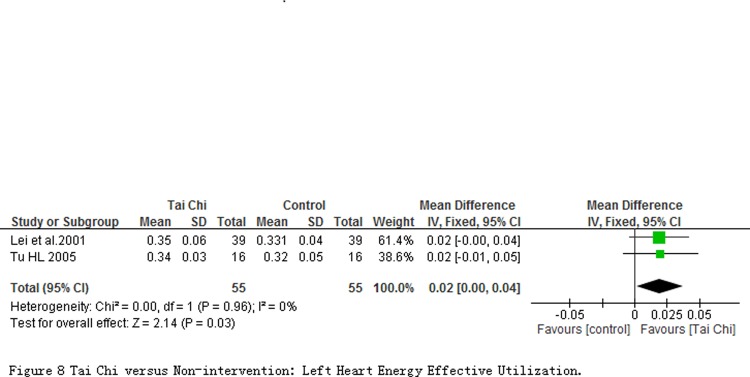
Tai Chi versus Non-intervention: Left Heart Energy Effective Utilization.

**Fig 9:**
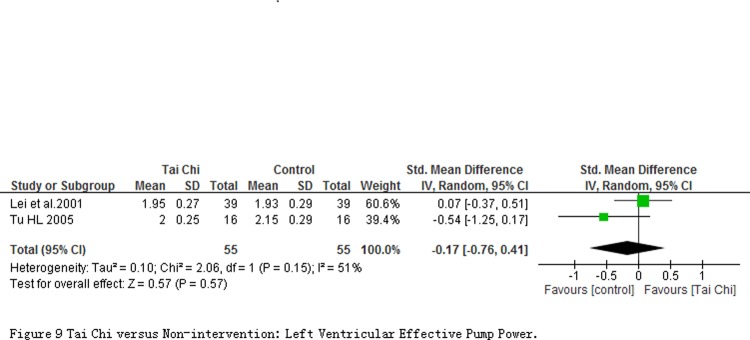
Tai Chi versus Non-intervention: Left Ventricular Effective Pump Power.

2 studies involving 110 participants measured outcomes about Myocardial oxygen consumption (HOV), Left heart energy effective utilization (EWK) and Left ventricular effective pump power (VPE) at quiet condition. Comparing to controls, the significant reduction on the HOV (MD = -4.11 mL/min; 95% CI-7.31 to-0.91; *P* = 0.01; Fig. 7) and increase on the EWK (MD = 0.02%; 95% CI 0.00 to 0.04; *P* = 0.03; Fig. 8) in participants with TC exercise were observed. But the significant changes was not found in the VPE outcome (MD = -0.03 Kg/n; 95% CI-0.14 to 0.07; *P* = 0.55; Fig. 9).

**Fig 10:**
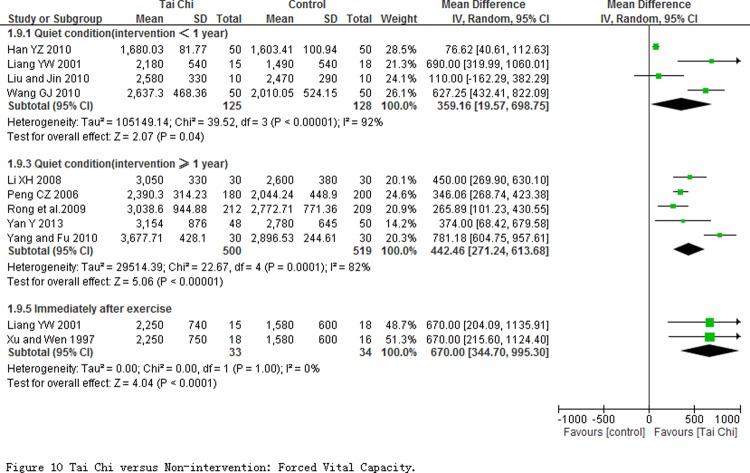
Tai Chi versus Non-intervention: Forced Vital Capacity.

**The effect of respiratory efficiency.** Forced vital capacity (FVC) was measured at quiet condition in 9 studies (involving in 1272 participants), and measured immediately after exercise in 2 studies with 67 participants (Fig. 10). Comparing to non-intervention, Subgroup analyses showed TC exercise had a mean of 359.16 mL improvement (95% CI 19.57 to 698.75, *P* = 0.04) for less than one year intervention, and a mean of 442.46 mL improvement (95% CI 271.24 to 613.68, *P* <0.0001) for more than one year intervention. But heterogeneity among studies was substantive, and *I* ^*2*^ value were 92%, 82% respectively. The heterogeneity in subgroup with intervention period less than one year was not obviously changed after sensitivity analysis was performed by removing one of any studies. But in subgroup with intervention period more than one year, the heterogeneity among studies took an obvious change from *I* ^*2*^ = 82% to 0% when sensitivity analysis was conducted by removing Yang and Fu 2010. Additionally, when measured immediately after exercise, the pooled result from 2 studies showed TC training significantly enlarged the FVC efficiently (MD = 670.00 mL; 95% CI 344.70 to 995.30; *P*<0.0001).

**Fig 11:**
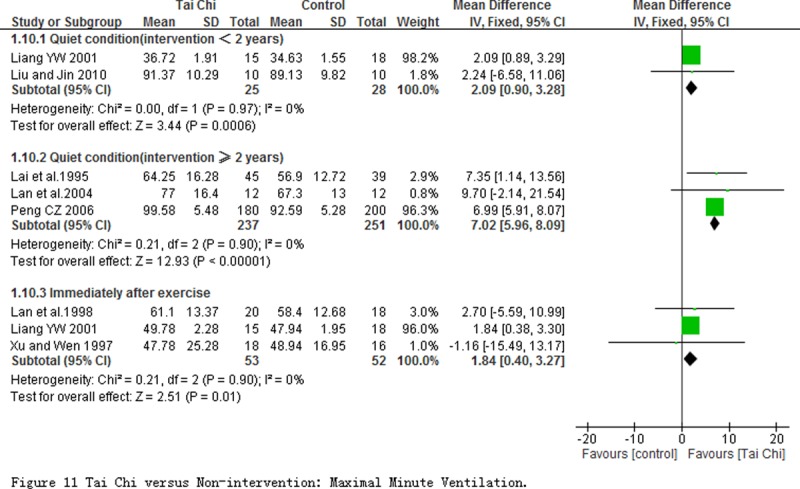
Tai Chi versus Non-intervention: Maximal Minute Ventilation.

5 studies measured the maximal minute ventilation (MMV) at the quiet condition. Comparing to non-intervention, the subgroup analyses on the basis of the different intervention period showed TC exercise had significant improvement to MMV regardless of intervention period for less than 2 years (MD = 2.09 L/min; 95% CI 0.90 to 3.28; *P* = 0.0006) or more than 2 years (MD = 7.02 L/min; 95% CI 5.96 to 8.09; *P*<0.00001). 3 studies immediately evaluated this outcome after exercise, and the pooled result showed a obvious improvement of MMV (MD = 1.84 L/min; 95% CI 0.40 to 3.27; *P* = 0.01) in participants with TC exercise comparing with those with non-intervention (Fig. 11).

**Fig 12:**
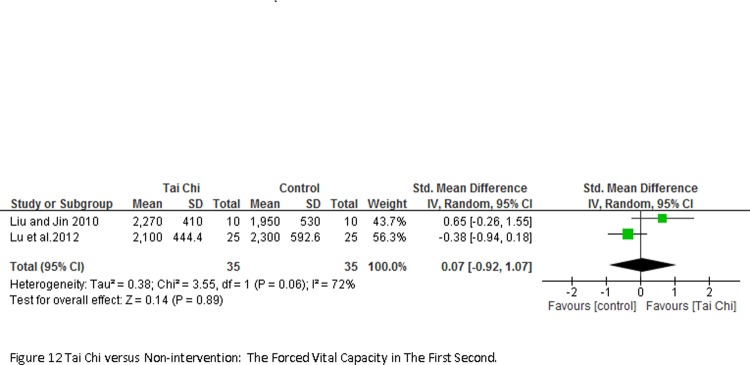
Tai Chi versus Non-intervention: The Forced Vital Capacity in The First Second.

2 studies involving 70 participants reported the forced vital capacity in the first second (FEV_1_) measured at quiet condition (Fig. 12). Result of meta-analyses showed no significant changes between TC exercise and control groups (SMD = -0.07; 95% CI-0.92 to 1.07; *P* = 0.89).

**Fig 13:**
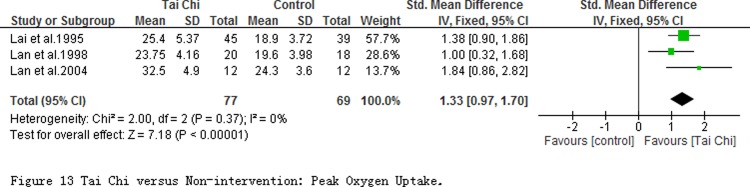
Tai Chi versus Non-intervention: Peak Oxygen Uptake.

The peak oxygen uptake (V·O_2_peak) at quiet condition was evaluated in 4 studies with 246 participants (Fig. 13), and the pooled result showed a significant increase in TC exercise group (SMD = 1.33; 95% CI 0.97 to 1.70; *P* < 0.00001) with no heterogeneity.

**Fig 14:**
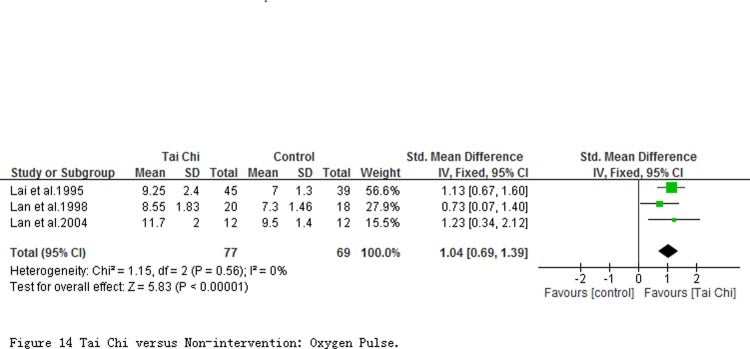
Tai Chi versus Non-intervention: Oxygen Pulse.

**The effect of cardiorespiratory endurance.** 3 studies (involving 146 participants, Fig. 14) reported the oxygen pulse (O_2_ pulse) at quiet condition. Comparing to non-intervention, the result of meta-analyses showed a significant improvement (SMD = 1.04; 95% CI 0.69 to 1.39; *P* < 0.00001) in participants with TC exercise.

**Fig 15:**
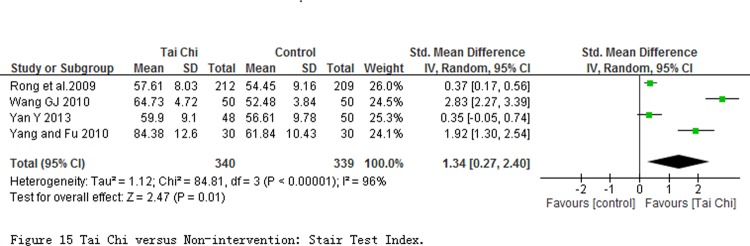
Tai Chi versus Non-intervention: Stair Test Index.

4 studies with 679 participants reported stair test index at quiet condition. The pooled result revealed a significant SMD for the effect of TC exercise on stair test index at quiet condition (SMD = 1.34; 95% CI 0.27 to 2.40; *P* = 0.01; Fig. 15). But heterogeneity among studies was substantive (*I* ^*2*^ = 96%, *P*<0.00001), and no obvious changes in heterogeneity by sensitivity analysis through removing any one of those studies.

### Adverse events

No included study reported the adverse events.

## Discussion

### Summary of Main Results

In this systematic review, 20 studies with 1783 healthy adults comparing TC exercise with non-intervention were included. The measured outcomes on CRF involved in cardiovascular efficiency, respiratory efficiency, and cardiorespiratory endurance. The review indicated that TC exercise might have a significant impact in improving cardiovascular efficiency by reducing resting blood pressure (SBP: SMD = -0.93; DBP: SMD = -0.94) and resting heart rate (SMD = -0.72), as well as enhancing stroke volume (MD = 0.44 mL/n) and cardiac output (MD = 0.32 L/min) at quiet condition, and HOV, EWK and VPE. There was substantial unexplained statistical heterogeneity observed in the resting blood pressure and resting heart rate, and small included studies in the HOV, EWK and VPE analysis, which suggested that the need for caution in interpreting these results. In addition, the present review also found significant improvement of TC exercise on respiratory function expressed by FVC, MMV, EFV_1_, VO_2peak_, and on cardiorespiratory endurance by measured stair test index and oxygen pulse. This review found no significant effect of Tai Chi exercise on VPE and EFV_1_. Nevertheless, these findings suggest that TC manifests promise as an optional exercise modality for the healthy adults to enhance CRF.

Tai Chi is a Chinese traditional mind-body exercise with a low to moderate exercise intensity [69]. The exercise intensity of TC mainly depends on its training style, posture and duration, and variation in training style and duration will result in substantial differences in exercise intensity [70]. In the present review, Yang style Tai Chi was used in six included studies, and other 14 studies did not report the definite style of TC practiced (only Taiji or Taijiquan). Furthermore, their training duration of TC varied greatly; the length of time ranged from 8 weeks to several years and frequency from 2 times to 7 times per week with 12 to 60 minute per time. These discrepancies may prevent to elucidate the potential applications for TC. Previous systematic reviews indicated that TC might be safe and effective in reducing blood pressure, improving aerobic capacity and exercise tolerance for patients with cardiovascular conditions and risk factors [71–72]. However, no previous review systematically assessed TC exercise for cardiorespiratory function regardless of healthy population or patients. A narrative review [73] demonstrated TC exercise had beneficial effects on outcomes of cardiorespiratory function including resting blood pressure, oxygen uptake (VO_2_), oxygen pulse, step index, and peak work rate in healthy older or patients with chronic disease. Another narrative review also suggested that Tai Chi exercise might have benefits to cardiovascular risk factors, such as hypertension, diabetes, dyslipidemia, poor exercise capacity, and endothelial dysfunction in patients with CVD, and indicated that Tai Chi exercise might promote cardiovascular health for patients with CVD [74]. However, these results are difficult to confirm the effect of TC for cardiorespiratory function owing to the unclear controls for evaluating the comparisons and unknown methodological quality in included studies.

### Limitations and Suggestions for Future Research

This review and meta-analysis included 20 studies and covered randomized controlled, non- randomized controlled, self-controlled trial and cohort study. And the conclusion can be, to a great extent, certified more comprehensively and accurately owing to the diversity of experiments design. Even though the meta-analysis has confirmed some promising conclusions, there is also a great deal of limitation. The review extracted a large amount of non-random controlled trails and cohort study as well as 3 self-control trails which had inherent imperfection with poor quality of methodology and high risk of bias. The trails were filled with multifarious interference factor, and the results of meta-analysis were triggered to reach high heterogeneity and low reliability. Furthermore, another primary and extremely important limitation of this review is the subgroup analysis. Some of subgroups with certain outcomes only included one study or two due to the limitation of eligible trails. Until more trails are available with identical outcomes, we didn’t evaluate accurately and broadly the effect of TC to CRF.

The increasing number of healthy community-dwellers was inclined to selecting TC as their fitness programs thanks to the free of limitation of field, instrument and gender. TC is regarded as a kind of historic civilization treasure in China, adhering to the simple dialectical ideology which harmonizes Yin-Yang, dynamic-static state and facilitate homeostasis body and spirit. Especially, TC is a moderate form of exercise which requires control of balance and weight [75], and coordination of movements [76]. In addition to those, TC also need muscles relaxed whilst concentrating on the specific mental attention and mix deep breathing regulation and meditation together [77–78]. And just because of those, TC might be the preliminarily recommended as a fitness program for the adults especially for the elderly. TC has been shown to be a kind of effective program to improve CRF [21] and health fitness [54]. However, the research for TC is not lucubrated and comprehensive so far. We have searched more than ten thousands of studies in Chinese and over one thousand in English up to October 2013. Most of studies about TC cover balance, hypertension, autonomic nerve, cardiovascular, lung disease and so on. There are several trials about the influence of TC on the CRF for the elderly. However, no one study with low risk has described all the indicators about the CRF. And more random, single-blind paralleled trails are need conducted randomization and allocation concealment with more standardized outcomes.

## Conclusion

The results of this review suggest that TC exercise is helpful for the healthy adults on improving the most outcomes of CRF, mainly on blood pressure, stroke volume, FVC, MMV, and VO_2peak_. However, concerning the low methodological quality, and the discrepancies of study design and TC training protocols in the included studies, the accurate conclusion can’t be drawn so far and these findings need to be interpreted cautiously. More larger-scale well-designed trails using standardized training protocols are needed till the specific and accurate conclusions can be perorated.

## Supporting Information

S1 File The detail of search strategy. (DOCX)

S2 File The detail of excluded studies. (DOCX)

S1 PRISMA Checklist (DOCX)
