# Supplementary material for: The Effect of Tai Chi Training on Cardiorespiratory Fitness in Healthy Adults: A Systematic Review and Meta-Analysis
Source: PLoS One. 2015 Feb 13;10(2):e0117360. doi: 10.1371/journal.pone.0117360 (PMC4332633; doi:10.1371/journal.pone.0117360)
Supplement: S1 File — (DOCX) [file pone.0117360.s001.docx]

**File S1: The detail of search strategy**

**PubMed SCI Cochrane**

#1:Taiji

#2:Tai Chi

#3:Chi, Tai

#4:Tai Ji Quan

#5:Ji Quan, Tai

#6:Quan, Tai Ji

#7:Tai-ji

#8:Taijiquan

#9:T'ai Chi

#10:Tai Chi Chuan

#11:1-10#/OR

#12:cardiopulmonary function

#13:maximal oxygen

#14:FVC

#15:Forced Vital Capacity

#16:gas exchange rate

#17:stroke volume

#18:VE minute ventilation

#19:minute respiratory volume

#20:EWK

#21:myocardial oxygen consumption

#22:HOV

#23:myocardial oxygen consumption index

#24:MOCI

#25:HOI

#26:maximal oxygen consumption

#27:FEK

#28:expansion coefficient of elasticity of blood vessels

#29:heart rate

#30:blood pressure

#31:oxygen pulse

#32:12-31#/OR

#33:control

#34:comparison

#35:controlled trial

#36:#33 OR #34 OR #35

#37：#11 AND #32 AND #36

**Embase**

#1 (tai-ji or tai chi or tai ji quan or ji quan, tai or quan, tai ji or tai ji or taijiquan or T'ai chi or tai chi chuan).mp. [mp=title, abstract, subject headings, heading word, drug trade name, original title, device manufacturer, drug manufacturer, device trade name, keyword]

#2 (FVC or maximal oxygen or cardiopulmonary function or Forced Vital Capacity or gas exchange rate or stroke volume or VE minute ventilation or minute respiratory volume or EWK or myocardial oxygen consumption or HOV or myocardial oxygen consumption index or MOCI or HOI or maximal oxygen consumption or FEK or expansion coefficient of elasicity of blood vessels or heart rate or blood pressure or oxygen pulse).mp. [mp=title, abstract, subject headings, heading word, drug trade name, original title, device manufacturer, drug manufacturer, device trade name, keyword]

#3 (comparison or "controlled trial" or control).mp. [mp=title, abstract, subject headings, heading word, drug trade name, original title, device manufacturer, drug manufacturer, device trade name, keyword]

#4 1 and 2 and 3

**维普**

检索式为：任意字段=太极 与 任意字段=心肺功能 与 任意字段=对照 与 范围=全部期刊(19篇)

**万方**

检索式为：主题：（[太极） * 主题：（心肺功能） * 对照 * Date:-2013 DBID:WF_QK](javascript:copyToClipboard() （16篇）

**CNKI**

中国学术期刊网络出版总库

检索式为：（主题=太极）AND（全文=心肺功能）AND（全文=对照）

[中国博士学位论文全文数据库](http://epub.cnki.net/grid2008/jump.aspx?url=http://acad.cnki.net/Kns55/loginid.aspx?uid=%7BUID%7D&p=Navigator.aspx?ID=CDFD)

检索式为：（主题=太极）AND（全文=心肺功能）AND（全文=对照）

[中国优秀硕士学位论文全文数据库](http://epub.cnki.net/grid2008/jump.aspx?url=http://acad.cnki.net/Kns55/loginid.aspx?uid=%7BUID%7D&p=Navigator.aspx?ID=CMFD)

检索式为：（主题=太极）AND（全文=心肺功能）AND（全文=对照）
