# Supplementary material for: The Effect of Tai Chi Training on Cardiorespiratory Fitness in Healthy Adults: A Systematic Review and Meta-Analysis
Source: PLoS One. 2015 Feb 13;10(2):e0117360. doi: 10.1371/journal.pone.0117360 (PMC4332633; doi:10.1371/journal.pone.0117360)
Supplement: S2 File — (DOCX) [file pone.0117360.s002.docx]

**File S2: The reasons of excluded studies**

| Title | Reason |
| --- | --- |
| 12-month Tai Chi training in the elderly: its effect on health fitness [45] | duplicate publication |
| Tai Chi Chih Acutely Decreases Sympathetic Nervous System Activity in Older Adults [46] | unqualified control intervention: Tai Chi versus healthy education |
| The effect of Tai Chi training on the function and component of physical health in the elderly [47] | incomplete statistical results: the lack of participants number |
